# Accelerated Muscle Deoxygenation in Aerobically Fit Subjects During Exhaustive Exercise Is Associated With the *ACE* Insertion Allele

**DOI:** 10.3389/fspor.2022.814975

**Published:** 2022-02-28

**Authors:** Benedikt Gasser, Martino V. Franchi, Severin Ruoss, Annika Frei, Werner L. Popp, David Niederseer, Silvio Catuogno, Walter O. Frey, Martin Flück

**Affiliations:** ^1^Departement für Bewegung und Sport – Universität Basel, Basel, Switzerland; ^2^Spinal Cord Injury Center, Balgrist University Hospital, Zurich, Switzerland; ^3^Department of Cardiology, University Hospital Zurich, University of Zurich, Zurich, Switzerland; ^4^Swiss Olympic Medical Center, Balgrist University Hospital, Zurich, Switzerland; ^5^Laboratory for Muscle Plasticity, University of Zurich, Balgrist Campus, Zurich, Switzerland

**Keywords:** cycling, aerobic metabolism, oxygen saturation, gene, exhaustive pedaling

## Abstract

**Introduction:**

The insertion/deletion (I/D) polymorphism in the gene for the major regulator of vascular tone, angiotensin-converting enzyme-insertion/deletion (ACE-I/D) affects muscle capillarization and mitochondrial biogenesis with endurance training. We tested whether changes of leg muscle oxygen saturation (SmO_2_) during exhaustive exercise and recovery would depend on the aerobic fitness status and the ACE I/D polymorphism.

**Methods:**

In total, 34 healthy subjects (age: 31.8 ± 10.2 years, 17 male, 17 female) performed an incremental exercise test to exhaustion. SmO_2_ in *musculus vastus lateralis* (VAS) and musculus gastrocnemius (GAS) was recorded with near-IR spectroscopy. Effects of the aerobic fitness status (based on a VO_2peak_ cutoff value of 50 ml O_2_ min^−1^ kg^−1^) and the ACE-I/D genotype (detected by PCR) on kinetic parameters of muscle deoxygenation and reoxygenation were assessed with univariate ANOVA.

**Results:**

Deoxygenation with exercise was comparable in VAS and GAS (*p* = 0.321). In both leg muscles, deoxygenation and reoxygenation were 1.5-fold higher in the fit than the unfit volunteers. Differences in muscle deoxygenation, but not VO_2_peak, were associated with gender-independent (*p* > 0.58) interaction effects between aerobic fitness × ACE-I/D genotype; being reflected in a 2-fold accelerated deoxygenation of VAS for aerobically fit than unfit ACE-II genotypes and a 2-fold higher deoxygenation of GAS for fit ACE-II genotypes than fit D-allele carriers.

**Discussion:**

Aerobically fit subjects demonstrated increased rates of leg muscle deoxygenation and reoxygenation. Together with the higher muscle deoxygenation in aerobically fit ACE-II genotypes, this suggests that an ACE-I/D genotype-based personalization of training protocols might serve to best improve aerobic performance.

## Introduction

Exercise pronouncedly elevates the energy expenditure of working skeletal muscle due to increased ATPase activity of the actin-myosin filaments and ion pumps (Rolfe and Brown, 1997). The resultant energy demand is met by enhanced aerobic combustion of organic substrates in mitochondria (Tonkonogi and Sahlin, 2002; Ryan et al., 2014); giving rise to the commonly observed oxygen deficit with exercise (Lukin and Ralston, 1962; Nioka et al., 1998). The implicated enhancement of mitochondrial respiration in contracting skeletal muscle can be readily, and indirectly, detected based on an increased signal for deoxygenated myoglobin/hemoglobin as measurable with near-IR spectroscopy (NIRS) (Nioka et al., 1998; Richardson et al., 2001; Perrey and Ferrari, 2018). The thereby observable decreases in muscle oxygen saturation (SmO_2_) are graded to the duration and intensity of exercise (Nioka et al., 1998; Chuang et al., 2002) and seem to be faster at the local, compared to the systemic (i.e., cardiopulmonary) level (Nioka et al., 1998; Grassi and Quaresima, 2016).

The changes in muscle oxygen saturation during intense prolonged exercise are dependent on the endurance capacity, respectively, aerobic fitness (Hoppeler et al., 1985). Thereby the general consensus is that endurance-trained individuals demonstrate elevated rates of oxygen desaturation with the onset of exercise (deoxygenation), compared to unfit subjects. Accordingly, an improved regain of oxygen saturation (reoxygenation) with the offset of exercise (Jones et al., 2017; Perrey and Ferrari, 2018).

Plausibly the alterations changes in oxygen saturation with on and offset of exercise are in a linear relationship to the aerobic capacity of skeletal muscle (Hoppeler et al., 1985; Jones et al., 2002; Tonkonogi and Sahlin, 2002; di Prampero, 2003; Fluck and Hoppeler, 2003; Flueck et al., 2010; Grassi and Quaresima, 2016). The capacity is set by the volume content of mitochondria which demonstrates itself considerable variability in relation to the maximal or peak oxygen uptake (peakVO_2_) (Hoppeler et al., 1985; Jones et al., 2002; Tonkonogi and Sahlin, 2002; di Prampero, 2003; Fluck and Hoppeler, 2003; Flueck et al., 2010; Grassi and Quaresima, 2016). Aside, vaso-regulatory factors modulate mitochondria-dependent muscle respiration by modulating the perfusion and consequently oxygen supply of recruited muscle fibers with the onset of contraction (Hoppeler et al., 1985; Zoll et al., 2002; Clifford and Hellsten, 2004; Roseguini et al., 2010; Korthuis, 2011; Hellsten and Nyberg, 2015).

Genetic influences on the regulation of blood flow with exercise are known to contribute to muscle energy metabolism (van Ginkel et al., 2015). Particularly, we have identified that a frequent insertion/deletion polymorphism in the gene that codes for angiotensin-converting enzyme (*ACE*) is associated with differences in blood flow with the onset of intense cyclic exercise, and oxygen-dependent mitochondria metabolism as well as with fluctuations in gene expression (Jackman et al., 2008; Dimitriou et al., 2010; Williams et al., 2017; Fluck et al., 2019). The implicated genetic mechanism is mediated by differences in the expression of the encoded ACE protease, which processes the main vasoconstrictor peptide, angiotensin 2, and the degradation of the vasodilatative kinin peptides (Jones et al., 2002). The presence of a 287-basepair insertion (the I-allele) in intron 16 of the *ACE* gene silences tissue expression of *ACE* transcripts, reducing ACE activity and angiotensin 2 levels in the blood; whereas its absence (i.e., deletion, D-allele) is associated with the inverse effects (Jackman et al., 2008; Vaughan et al., 2013; Mathes et al., 2015; van Ginkel et al., 2016; Fluck et al., 2019). *ACE* I-allele carriers demonstrate exaggerated gains in the volume density of mitochondria in knee extensor muscle with repeated endurance training (Vaughan et al., 2013; Fluck et al., 2019); indicating that ACE-insertion/deletion (I/D) genotype-associated differences in aerobic metabolism develop with repeated sessions of endurance exercise.

Importantly, healthy ACE-DD genotypes demonstrate a lower muscle capillarization and a reduced capillary perfusion with endurance exercise than *ACE* I-allele carriers (van Ginkel et al., 2015; Valdivieso et al., 2017). Collectively, the observed ACE-I/D-related response to acute and repeated endurance exercise indicated either an elevated vasoconstriction or a lower potential for vasodilatation, in *ACE* D-allele carriers; giving rise to a lowered capacity for oxygen delivery to recruited skeletal muscles during and after exercise (Buikema et al., 1996; O'Donnell et al., 1998; van Dijk et al., 2000; Williams et al., 2000; Woods et al., 2002; Fluck and Hoppeler, 2003; Flueck et al., 2010).

Whether the reported influence of the ACE-I/D gene polymorphism on mitochondria and capillary processes and connected metabolic processes of skeletal muscle results in differences in muscle oxygen saturation during intense exercise is not known. Toward this end we tested the hypotheses, the ACE-I/D polymorphism is associated with (i) an accelerated and more extensive deoxygenation in aerobically fit subjects I-allele carriers compared to non-I-allele carriers, and whether (ii) the reoxygenation is accelerated in *ACE* I-allele carriers and may be further accelerated by a good fitness state.

## Methods

### Study Design

This study had a cross-sectional design in which the subjects performed a ramp test to determine VO_2peak_, Peak Power Output (PPO) and SmO_2_ in a knee extensor muscle (VAS) and an ankle extensor muscle (GAS). Prospective power analysis on the association of the ACE-I/D genotype with aerobic processes in skeletal muscle indicated that a total number of 24 replicas (distributing equally according to each of the four combinations of genotype and fitness type) is sufficient enough to reveal statistically significant associations between the ACE-I/D genotype × fitness state and differences in muscle deoxygenation (Supplementary Table 1). As the average age of participants was 31.81 ± 10.18 years in line with standard guidelines subjects were assigned as being aerobically fit or unfit based on whether their respective VO_2peak_ met the criteria of being above or below 50 mL·min^−1^·kg^−1^ (Valdivieso et al., 2017; ACSM, 2021). ACE I/D gene polymorphism genotyping was performed retrospectively.

### Subjects/Ethics

A total of 34 recreational active healthy subjects, i.e., 17 women and 17 men, participated in this study. Subject recruitment was by word-of-mouth and a public announcement with flyers in the professional or private environment of the research group. All subjects volunteered after their self-assessment that they met the requirements of presenting a good health and an age between 20 and 70 years. Upon the provision of an informed consent, the further specific inclusion criterium of an unobtrusive cardiovascular system was verified based on an inconspicuous ECG during exercise in a ramp test on a cycle ergometer. The exclusion criteria comprised evidence for a relevant valvular heart disease, arterial hypertension (blood pressure at rest > 140/90 mm Hg), arrhythmogenic cardiomyopathy, smoker, drug, or alcohol disease, known or suspected non-compliance with the protocol, or a contraindication for ethical reasons. All the 34 participating volunteers qualified for the inclusion in this study. The study has been approved by the Ethics Committee of the Canton of Zurich. All the investigations were conducted in accordance with the ethical standards of the Declaration of Helsinki of 1964.

### Ramp Test

Subjects performed a test of incremental exercise on an electrically braked cycle ergometer (Ergoselect 200, Ergoline, Bitz, Germany, UK) being accompanied with spiroergometric and NIRS measurements. Prior to the test, anthropometric data (height and body mass) were collected, and the body mass index (BMI) was calculated. A resting ECG was recorded and verified by a physician to ensure that the subjects did not demonstrate a counter-indication to conduct the ramp test.

The test protocol was conducted in an air-conditioned laboratory at a standardized temperature of 20°C according to a modified version of a published protocol (Whipp et al., 1981). In brief, the test started with a 3 min period of rest, when subjects sat still on the cycle ergometer while maintaining a normal breathing pattern. Subsequently, subjects started pedaling at an initial power (75 W for women and 100 W for men). Target power was increased every 20 s (18 W·min^−1^ for women and 30 W·min^−1^ for men). The subjects were asked to keep a constant self-chosen pedal cadence throughout the test (optimally between 70 and 100 rpm). The test was stopped when the subjects experienced volitional exhaustion and/or were not able to maintain the target pedal cadence. Subsequently, recordings continued during a period of 8 min, when subjects rested in a seated position on the cycle ergometer.

Pulmonary gas exchange (oxygen uptake) was measured after proper calibration through a breath-by-breath spiroergometry system (MetaLyzer^®^ 3B-R2, CORTEX Biophysics, Leibzig, Germany). VO_2peak_ and PPO were defined as the last achieved, and peak, values before exhaustion manifested. To non-invasively measure SmO_2_ during the ramp test, a muscle NIRS monitor (Moxy, Fortiori Design LLC, Minnesota, USA) was used as established (Fitze et al., 2019). The device uses four different light sources covering wavelengths in the range of 630 to 850 nm and a modified Beer–Lambert law to perform measurements of SmO_2_. For measurements, two NIRS monitors were positioned on specific sites of the skin that were shaved using a disposable razor (Gallant, Dynarex, Orangeburg, USA) and cleaned with an alcohol swab (Webcol™, Covidien™, Dublin, Ireland). One monitor was placed on the lower third of the VAS in the middle of the muscle belly on the left leg of the subjects. The other monitor was placed on the GAS, onto a fictive line between the Malleolus medialis and medial plateau of the tibia. Sensors were attached using the recommended tape (Moxy Adhesive Attachments, Fortiori Design LLC, Minnesota, USA). In order to protect the NIRS device from ambient light, it was covered with an adhesive non-woven fabric (Hypafix^®^, BSN medical, Hamburg, Germany). Recording started synchronized with the start of the ramp test (with 3 min of rest).

### Genotyping

Buccal swabs were collected with an ear-bud, air-dried in a laboratory fume cupboard (Secuflow 1500, Waldner, Wangen, Germany, UK) for 2 h and then stored at +4°C. Subjects were told not to consume any food or to drink in the 30 min prior to sample collection.

Deoxyribonucleic acid extraction was performed according to a commercially available protocol (QIAamp^®^ DNA Mini Kit, Qiagen, Hilden, Germany, UK). In brief, the cotton swab was separated from the stick with scissors, followed by incubation steps in a 2 ml microcentrifuge tube to degrade contaminating ribonucleic acids, lyse the cells with QIAGEN^®^ proteinase K, and enrich the contained genomic DNA with QIAamp Mini spin columns with the help of an air-cooled microcentrifuge (Prism™, Labnet International, Edison, USA). The resulting sample (150 μl) elute was stored at +4°C until genotyping was performed.

Genotyping was carried out by polymerase chain reactions in 48 well-plates followed by high-resolution melt analysis using a real-time PCR system (Eco™, illumina^®^, San Diego, USA) according to the instructions. The reaction mix per well-included for each sample 2 μl of the DNA solution, 1 μl distilled H_2_O, 1 μl of MgCl_2_ (25 mmol), 5 μl of KAPA HRM FAST Master Mix (2×) and 1 μl of the I- or D-allele-specific primer mix (2 μmol). The primer mix for the detection of the 66 bp amplicon, which is specific for the I-allele, contained the primer ACE2 (5′-TGGGATTACAGGCGTGATACAG-3′) and the primer ACE3 (5′-ATTTCAGAGCTGGAATAAAATT-3′). The primer mix for the detection of the 83 bp amplicon, which is specific to the D allele, contained primers ACE1 (5′-CATCCTTTCTCCCATTTCTC-3′) and ACE3 (5′-ATTTCAGAGCTGGAATAAAATT-3′). The sealed plate was centrifuged to remove any bubbles and was submitted to a standardized thermal protocol as published (Valdivieso et al., 2017). Genotype analysis was carried out using a genetic variation analysis software (EcoStudy Version 5.0, Illumina^®^, San Diego, California, USA). The respective genotype was verified based on the presence of an allele-specific melting curve for the amplified products in the respective PCR reactions, as established by microsequencing of PCR reactions from reference samples at a commercial provider (Microsynth, Balgach, Switzerland, UK) (Valdivieso et al., 2017).

### Data Processing

**Figure 1:**
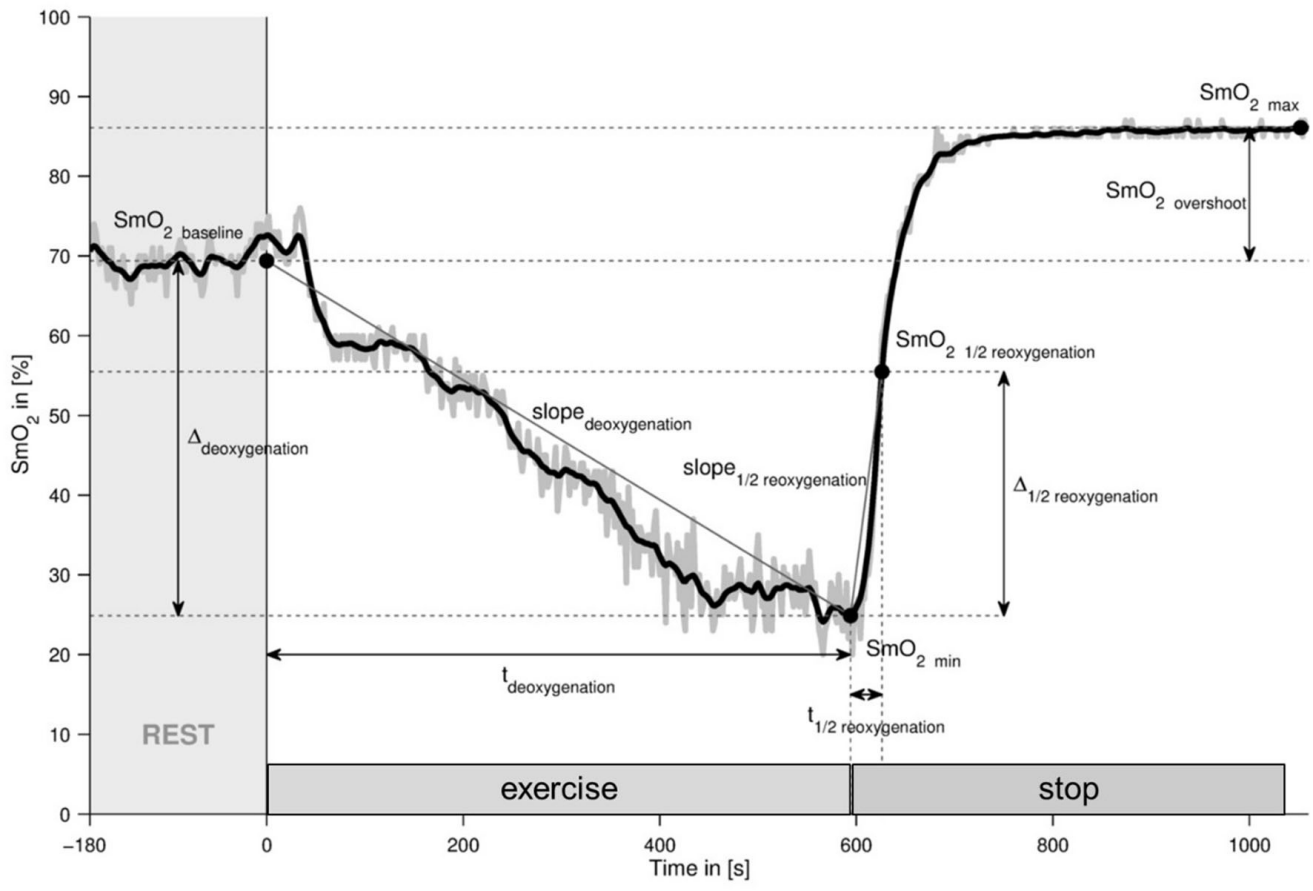
Representative example of the recorded SmO_2_ course during the ramp test. Line graph visualizing the raw (gray) and processed data (black) and the derived parameters. SmO_2_, muscle oxygen saturation; t, time; min, minimum; max, maximum. The extracted parameters are declared.

A representative example of the recorded and processed timeline of SmO_2_ during the ramp test, as well as the extracted parameters, is shown in Figure 1. Data pre-processing and analysis were performed as previously described using a data processing program (MATLAB 2015a, The Mathworks, Natick, USA) (Fitze et al., 2019). In short, SmO_2_ data were filtered using a 2nd order zero-phase shift Butterworth low-pass filter with a cutoff frequency of 0.03 Hz. Extraction of the values for relevant parameters was performed as previously described: SmO_2baseline_ was declared as the mean value of the t3-min prerest period. The minimum SmO_2_ value during the ramp test (SmO_2min_) was determined based on the last local minimum of the filtered SmO_2_ prior to reoxygenation. Δ_deoxygenation_ was set as the difference between SmO_2baseline_ and SmO_2_ min. t_deoxygenation_ was the time from the beginning of the ramp test until SmO_2min_ was reached. slope_deoxygenation_ was calculated of the values of Δ_deoxygenation_ over t_deoxygenation_. SmO_2max_ was defined as the highest value achieved within the period between the start of reoxygenation and test termination. SmO_21/2reoxygenation_ was defined as 50% of the difference between SmO_2max_ and SmO_2min_. Δ_1/2reoxygenation_ was the difference between SmO_21/2reoxygenation_ and SmO_2min_. t_1/2reoxygenation_ was defined as the time between SmO_2min_ and SmO_21/2reoxygenation_. Slope _1/2reoxygenation_ was calculated using Δ_1/2reoxygenation_ over t_1/2reoxygenation_. SmO_2overshoot_ represented the difference between SmO_2max_ and SmO_2baseline_. Concerning the assumption of a linear process over the ramp protocol before exhaustion of SmO_2_ and Power a high correlation was detected (*R* = −0.981 ± 0.214). For the display of the average course of SmO_2_ in VAS and GAS during the ramp test, the recorded raw values were averaged over 9 s intervals, and the mean and SE over all 34 samples calculated for each time point/9 s interval. For the values from the exercise phases, the “time coordinates” were scaled to the duration of a reference data set for a study participant which ceased pedaling nearest to the duration of the lower 25% quartile of t_deoxygenation_, i.e., 358 s.

### Statistical Analysis

An online calculator was used to determine whether the observed genotype frequency is consistent with the Hardy–Weinberg equilibrium (Rodriguez et al., 2009). Prospective and retrospective power analyses were conducted with G-Power (version 3.1.9.6, http://www.gpower.hhu.de/) and the Statistical Package for the Social Sciences (SPSS version 23, IBM, Armonk, USA), respectively. Variance homogeneity for sub-samples was analyzed with Levene Test, whereby only for t_1/2reoxygenation_ (*p* = 0.027) variance inhomogeneity was deduced. Therefore, a multivariate ANOVA was used to assess the effects of the ACE I/D genotype and aerobic fitness status, and their interaction, and the influence of the muscle type and gender, on parameters of muscle oxygenation (SmO_2_) during the ramp test. A co-dominant genetic model was applied to calculate the effects of the ACE I/D genotype, i.e., carriers vs. non-carriers of the D-allele. A *post-hoc* test of the least significant difference was applied to localize effects. Statistical analyses and graphical presentations were calculated with SPSS (SPSS version 23, IBM, Armonk, USA) and assembled for presentation using MS-Office Excel and Powerpoint (Microsoft Office Professional Plus 10, Kildare, Ireland, UK). Significance was declared depending on whether a *p*-value below 0.05 was met.

## Results

### Subjects Characteristics

Table 1 summarizes selected physiological and characteristics of the 34 volunteers per genotype and fitness status. Aerobically fit subjects demonstrated on average a 14.9 ml O_2_ min^−1^ kg^−1^ higher specific VO_2_ peak and a 112.6 W higher PPO than the unfit subjects. Body mass, BMI, height, and weight did not differ between the aerobically fit and unfit subjects.

**Table 1:** Physiological characteristics.

**Group**	***n***	**Age**	**Body mass**	**Height**	**BMI**	**VO2peak**	**PPO**
		**[years]**	**[kg]**	**[m]**	**[kg m** ^**−2**^ **]**	**[mL·min-1·kg** ^**−1**^ **]**	**[W]**
All	34	31.81 ± 10.18	69.34 ± 10.43	173.00 ± 8.40	23.08 ± 2.39	46.03 ± 9.61	298.26 ± 79.29
Aerobically unfit	20	32.07 ± 12.09	69.00 ± 10.80	170.60 ± 7.63	23.61 ± 2.55	39.89 ± 7.32	251.90 ± 61.09
Aerobically fit	14	31.43 ± 7.00	69.83 ± 10.26	176.43 ± 8.53	22.31 ± 1.98	54.79 ± 3.94	364.50 ± 49.75
D-allele carriers	25	32.17 ± 10.71	67.67 ± 10.46	172.92 ± 9.33	22.51 ± 1.96	46.42 ± 9.60	295.76 ± 80.51
D-allele non-carriers	9	30.81 ± 9.02	73.99 ± 9.35	173.22 ± 5.49	24.66 ± 2.87	44.94 ± 10.14	305.22 ± 80.09
Unfit D-allele carriers	14	31.97 ± 12.91	67.43 ± 10.51	170.36 ± 8.60	23.11 ± 1.91	39.75 ± 6.88	249.36 ± 68.56
Unfit D-allele non-carriers	6	32.30 ± 11.04	72.67 ± 11.55	171.17 ± 5.31	24.78 ± 3.58	40.22 ± 8.97	257.83 ± 43.57
Aerobically fit D-allele carriers	11	32.41 ± 7.65	67.97 ± 10.91	176.18 ± 9.58	21.74 ± 1.81	54.90 ± 4.28	354.82 ± 50.95
Aerobically fit D-allele non-carriers	3	27.84 ± 1.03	76.63 ± 0.55	177.33 ± 3.51	24.40 ± 0.82	54.40 ± 3.02	400.00 ± 26.46
Aerobic fitness	*p*	0.644	0.601	0.084	0.339	<0.001	<0.001
	η2	0.007	0.009	0.096	0.031	0.516	0.486
Genotype	*p*	0.626	0.114	0.772	0.022	0.995	0.258
	η2	0.008	0.081	0.003	0.163	0.000	0.042
Aerobic fitness	*p*	0.574	0.691	0.960	0.590	0.853	0.436
× genotype	η2	0.011	0.005	0.000	0.010	0.001	0.020

*Values represent mean ± SD of the measurements for the studied 34 subjects, as well as the p-values and effect sizes (η2) and observed power, for the effect of ACE-I/D genotype and aerobic fitness status, and their respective interaction. ANOVA with post-hoc test of Fisher. Underlined p-values were deemed to reflect significant effects*.

The studied population was found to stand in Hardy-Weinberg equilibrium (*p* = 0.261). The ACE-I/D genotype was not associated with differences in VO_2_peak or PPO independent of whether assessed as single effect (*p* = 1.00, *p* = 0.26) or interaction effect with the aerobic fitness state (*p* = 0.85, *p* = 0.44; Table 1). BMI was 2.2 kg/m^2^ higher in non-carriers than carriers of the *ACE* D-allele.

### Muscle Oxygen Saturation During the Ramp-Incremental Pedaling Exercise

Figure 1 visualizes an example of the alterations in SmO_2_ in *musculus vastus lateralis* during the ramp-incremental pedaling exercise. SmO_2_ was fairly stable during the first phase of rest (SmO_2_ baseline) and then fell with the onset of contraction (deoxygenation) at an average rate of −0.060 ± 0.028% SmO_2_ s^−1^ to a minimal value (SmO_2_ min) until voluntary exhaustion manifested. SmO_2_ rapidly recovered with a rate of 0.736 ± 0.459% SmO_2_ s^−1^, to, or above, baseline values with the cessation of exercise (reoxygenation).

**Figure 2:**
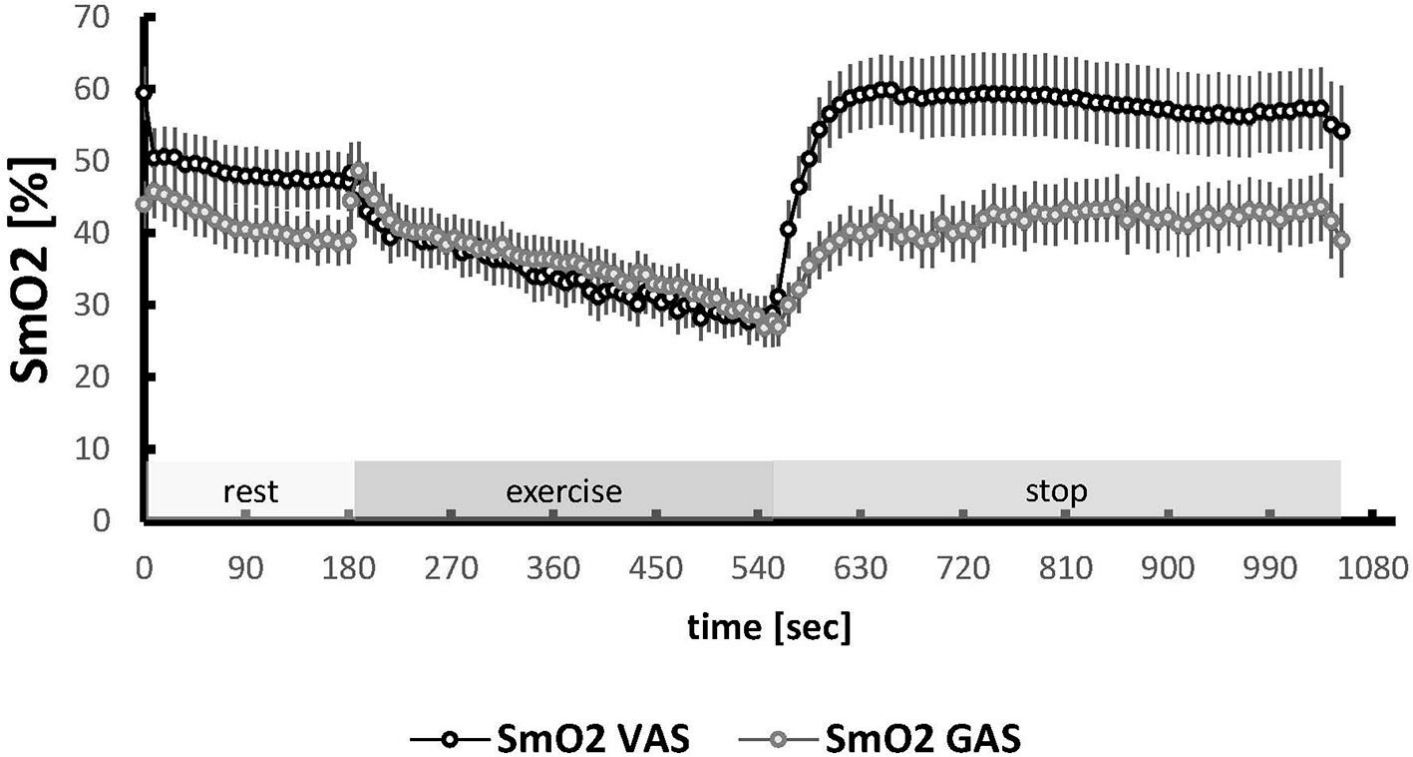
Oxygen saturation in VAS and GAS during the ramp test. Line graph visualizing the mean + SE (circle and vertical bars) of values for SmO_2_ in VAS and GAS during the course of the ramp test. The three phases of the test are indicated. Values were averaged to each 9 s interval for the measures from the 34 subjects. The time coordinates for the values during the exercise phase were scaled for each subject to the duration of a reference data set.

Muscle deoxygenation was similar between VAS and GAS muscle (*p* = 0.321, Table 2; Figure 2). Muscle type differences resolved for parameters of reoxygenation (Δ1/2 reoxygenation, SmO_2_ max, SmO2 overshoot; Supplementary Figure 1), and SmO_2_ at baseline (59.9 vs. 52.9%; *p* = 0.004), all being higher in VAS than GAS.

**Table 2:** Association between fitness status and angiotensin-converting enzyme-insertion/deletion (ACE-I/D) genotype on parameters of muscle oxygen kinetics during the ramp test of cycling exercise.

**Phase**	**Parameter**	**Statistical size**	**Fitness**	**Genotype**	**Fitness × genotype**	**Muscle**	**Muscle × fitness**	**Muscle × genotype**	**Fitness × genotype**
Rest	SmO_2baseline_	*p*-value	0.018	0.027	0.151	0.004	0.355	0.812	0.305
		η^2^	0.090	0.079	0.034	0.123	0.014	0.001	0.018
Exercise	SmO_2min_	*p*-value	≤ 0.001	0.597	0.098	0.052	0.208	0.031	0.296
		η^2^	0.240	0.005	0.045	0.058	0.026	0.075	0.018
Exercise	Δ_deoxygenation_	*p*-value	≤ 0.001	0.141	0.020	0.321	0.891	0.174	0.920
		η^2^	0.302	0.036	0.087	0.015	0.000	0.031	0.000
Exercise	t_deoxygenation_	*p*-value	0.011	0.174	0.151	0.586	0.834	0.519	0.472
		η^2^	0.104	0.031	0.034	0.005	0.001	0.007	0.009
Exercise	Slope_deoxygenation_	*p*-value	0.005	0.598	0.006	0.175	0.902	0.306	0.575
		η^2^	0.125	0.005	0.118	0.029	0.000	0.017	0.005
Stop	SmO_2max_	*p*-value	0.013	0.364	0.357	≤ 0.001	0.526	0.945	0.451
		η^2^	0.098	0.014	0.014	0.674	0.007	0.000	0.009
Stop	Δ1/2 _reoxygenation_	*p*-value	≤ 0.001	0.705	0.101	≤ 0.001	0.105	0.131	0.765
		η^2^	0.270	0.002	0.044	0.392	0.043	0.038	0.001
Stop	t1/2 _reoxygenation_	*p*-value	0.262	0.818	0.571	0.123	0.822	0.639	0.828
		η^2^	0.021	0.001	0.005	0.037	0.001	0.004	0.001
Stop	Slope_1/2reoxygenation_	*p*-value	0.031	0.598	0.365	0.823	0.682	0.607	0.592
		η^2^	0.076	0.005	0.014	0.001	0.003	0.004	0.005
Stop	SmO_2overshoot_	*p*-value	0.838	0.273	0.687	≤ 0.001	0.182	0.793	0.842
		η^2^	0.001	0.020	0.003	0.473	0.030	0.001	0.001

*Values refer to the calculated level of statistical significance (p) and effect size (η^2^) for the effect of the aerobic fitness state, ACE-I/D genotype (ACE-II vs. ACE-ID/ACE-DD), and muscle type on assessed parameters of deoxygenation and reoxygenation in the leg muscles of the studied 34 subjects. P-values that were deemed statistically significant (i.e., p < 0.05) are underlined. ANOVA with post-hoc test of least significant difference*.

### Aerobic Fitness Affects Muscle Deoxygenation and Reoxygenation During Ramp-Incremental Exercise

**Figure 3:**
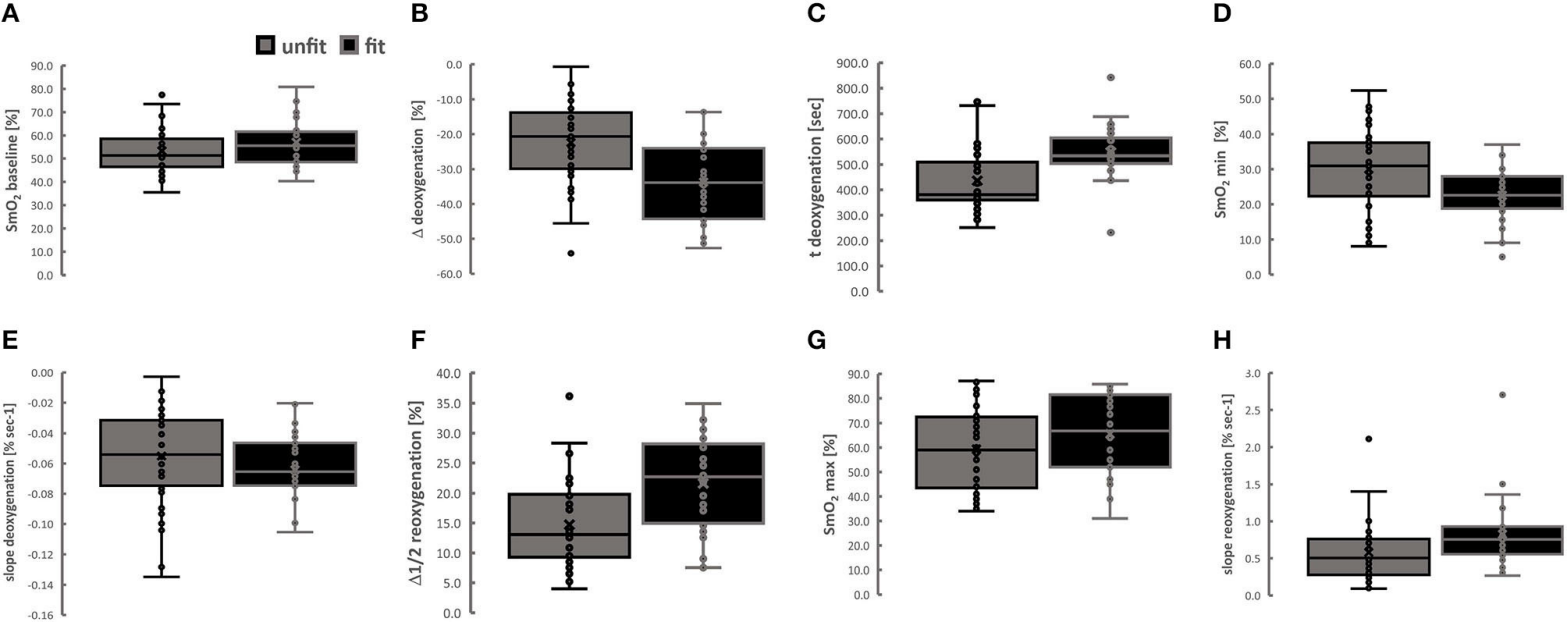
Aerobic fitness state affects the kinetics of muscle oxygenation during exercise. Box Whisker plots (Box, 5 and 95% confidence intervals; Whisker, minima-maxima; central line, median; circle, individual values) for kinetic parameters of muscle deoxygenation **(A–E)** and reoxygenation **(F–H)** in VAS and GAS combined. Lines connect conditions demonstrating significant differences at *p* < 0.05. ANOVA for the factor “aerobic fitness” with *post-hoc* test of Fisher for the least significant difference.

Parameters that characterized the deoxygenation and reoxygenation kinetics in the two-leg muscles with ramp-incremental pedaling exercise demonstrated associations with aerobic fitness (Table 2). Figure 3 depicts the differences for the corresponding effect of the aerobic fitness status for the combined values for both the leg muscles.

The values for SmO_2_ min (25.6%), Δ_deoxygenation_ (59.1%) and the slope_deoxygenation_ (22.1%), were lower in aerobically fit compared to unfit subjects, when the t_deoxygenation_ was higher in fit than unfit subjects (+24.2%). Conversely, the values for Δ_1/2reoxygenation_ (44.2%), slope _1/2reoxygenation_ (+39.0%) and SmO_2_ max (+9.4%) were higher in aerobically fit than unfit subjects.

When assesses separately for VAS and GAS, the values for SmO_2_ min (24.8 vs. 35.1%, 22.8 vs. 28.9%), Δ_deoxygenation_ (−34.9 vs. −21.7%, −31.1 vs. −19.8%) and the slope_deoxygenation_ (i.e., −0.065 vs. −0.054% SmO_2_ s^−1^, −0.056 vs. −0.045% SmO_2_ s^−1^) were all lower in aerobically fit compared to unfit subjects. Conversely, the values for Δ_1/2reoxygenation_ (27.8 vs. 18.4%, 15.5 vs. 11.6%) were higher in aerobically fit than unfit subjects. Additionally, the values for baseline SmO_2_ min in GAS (53.9 vs. 48.7%), SmO_2_ max in VAS (80.2 vs. 71.8%) and t_deoxygenation_ in VAS (543.7 s vs. 430.3 s) were higher in fit than unfit subjects.

### Differences in Muscle Deoxygenation During Ramp-Incremental Exercise Are Associated With the Interaction Between Aerobic Fitness × ACE-I/D Genotype

Baseline values for SmO2, alone, were associated with the ACE-I/D genotype (*p* = 0.027, Table 2). The values of two kinetic parameters resuming muscle deoxygenation, i.e., Δdeoxygenation and slope deoxygenation, demonstrated interactions between the aerobic fitness status and the ACE-I/D genotype (Table 2).

**Figure 4:**
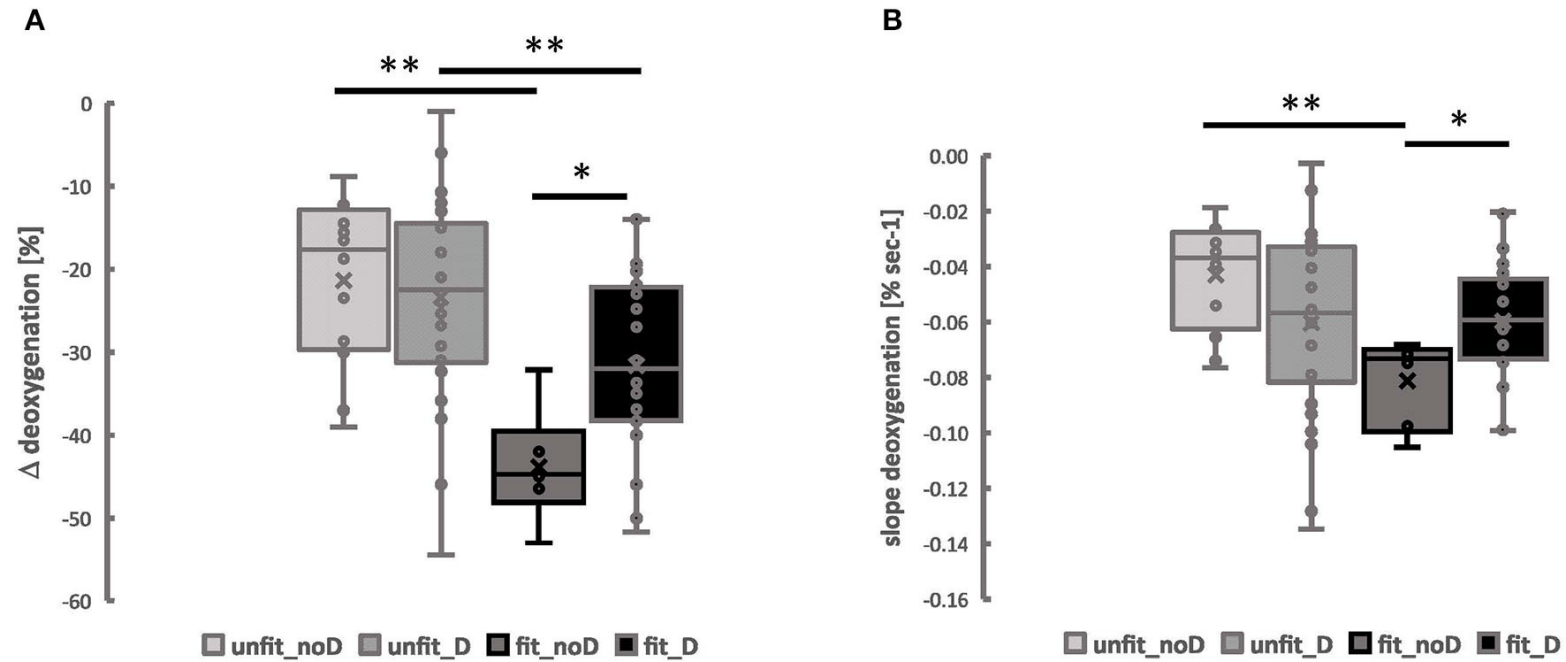
Muscle deoxygenation during exercise is associated with the interaction between the aerobic fitness state and the angiotensin-converting enzyme-insertion/deletion (ACE-I/D) genotype. Box Whisker plots for the Δ_deoxgenation **(A)** and the slope of deoxygenation **(B)** in VAS and GAS combined in the function of the aerobic fitness status and the ACE-I/D genotype. Lines connect conditions demonstrating significant differences at *p < 0.05 and **p < 0.01. ANOVA for the factor “aerobic fitness” with *post-hoc* test of least significant difference.

For both parameters, the interaction was localized to the lowest values in fit non-carriers of the *ACE* D-allele (Figure 4). For Δdeoxygenation, the values in the fit ACE-II genotypes were 29.4% lower than in fit non-carriers of the D-allele and 56.4% lower than in unfit ACE-II genotypes. Alike for the slope deoxygenation, the values in fit ACE-II genotypes were 52.5% lower than in unfit ACE-II genotypes and 31.3% lower than in fit carriers of the D-allele.

The interaction effect of aerobic fitness status and the ACE-I/D genotype for Δdeoxygenation and slope_deoxygenation_ was not affected by the muscle type (*p* = 0.920, *p* = 0.575), and sex (Supplementary Table 2), where Δdeoxygenation and slope_deoxygenation_ in VAS and GAS were the lowest in the fit ACE-II genotypes (Supplementary Figure 2).

Supplementary Table 3 depicts the average values for the observed parameters of deoxygenation and reoxygenation in both studied leg muscles for each genotype and fitness status.

## Discussion

The aim of this study was to investigate whether a NIRS-based measure of the balance between supply and use of oxygen during exhaustive pedaling exercise is associated with the ACE-I/D genotype and stands in dependence of the fitness state (Casey and Joyner, 2015; Ross et al., 2019). The mechanism underpinning muscle deoxygenation involves local reactions in skeletal muscle, such as contraction-induced elevations in capillary perfusion and an increased mitochondrial respiration (Badtke, 1987; Zoll et al., 2002; Clifford and Hellsten, 2004; Korthuis, 2011; Grassi and Quaresima, 2016).

The observed higher slopes and minima/maxima of muscle oxygen saturation during and after exhaustive ramp exercise are in line with previous observations on the enhanced and accelerated capacity for muscle deoxygenation of aerobically fit individuals during exhaustive pedaling exercise and subsequent reoxygenation with the offset of pedaling (Figure 2) (Ding et al., 2001; Brizendine et al., 2013; Casey and Joyner, 2015; Perrey and Ferrari, 2018; Ross et al., 2019). These observations support the view that NIRS-based measures of oxygen saturation are a proxy of mitochondrial activity (Pilegaard et al., 2002; Ryan et al., 2014). The resulting difference in the proxy for the local respiratory capacity between fit and unfit subjects indicated that this study would allow to identify whether the hypothesized association between the ACE-I/D genotype and kinetic aspects of oxygen saturation with exhaustive muscle work would depend on the aerobic fitness state.

Based on previous observations on ACE-I/D associated differences on angiotensin-modulated vasoconstriction (Korthuis, 2011; van Ginkel et al., 2015, 2016; Valdivieso et al., 2017), variability in exercise-induced changes in muscle oxygen saturation during the exhaustive type of pedaling exercise were expected to be associated, with the ACE-I/D genotype and the aerobic fitness status. We observed a 1.5-fold increased muscle deoxygenation in the aerobically fit than unfit subjects (Figure 3). Accordingly, the identified slopes of deoxygenation during exhaustive ramp exercise emphasized that this difference was due to an accelerated rate of deoxygenation for both investigated leg muscles in the aerobically fit compared to the unfit subjects, i.e., VAS: −3.5 vs. 2.9% per minute; GAS: −4.1 vs. −3.3% per minute). The slope 1/2 reoxygenation was overall 1.5-fold enhanced in aerobically fit compared to unfit subjects relating to the reportedly 2-fold faster recovery rates of oxygen consumption in *musculus vastus lateralis* of endurance athletes than inactive controls (Brizendine et al., 2013).

We explain the former observations in terms of the model that the precipitous drop of muscle oxygen saturation with contraction is due to enhanced oxygen consumption in mitochondria, which is not matched by the increase in muscle perfusion (Baker et al., 2010; Jones et al., 2017). Conversely, muscle reoxygenation postexercise arises because the respiratory activity of mitochondria levels off, while muscle perfusion continues to be elevated due to the fact that muscle capillaries remain maximally perfused for a longer time (Egginton and Hudlicka, 1999; Korzeniewski, 2003; Clifford and Hellsten, 2004). Anatomical factors that modify quantitative aspects of muscle oxygen saturation, such as the content of mitochondria and the capillarization, are an integral part of the underpinning processes. Accordingly, as muscle oxygen saturation is related to mitochondrial respiratory capacity (Ryan et al., 2014), the increased muscle deoxygenation and steeper negative slope of deoxygenation with pedaling exercise in the aerobically fit subjects would be explained by the functionally more developed capacity for mitochondrial respiration due to typical increases in mitochondrial volume density in trained skeletal muscles (Hoppeler et al., 1985). By contrast, the increased slope of reoxygenation for *Musculus vastus lateralis* during recovery from exhaustive exercise in the aerobically fit compared to the unfit individuals would be driven by the total capacity for capillary perfusion because the arterioles are in a fully dilated state in the recruited knee extensor muscles (Egginton and Hudlicka, 1999; Clifford and Hellsten, 2004). This suggestion would be supported by the observation that the estimated rate of reoxygenation was 9–15-fold accelerated in the studied leg muscles for both the fit and unfit subjects, compared to the corresponding rate of deoxygenation. Consequently, our results imply that capacitive differences in capillary perfusion exist between the aerobically fit and unfit participants of our investigation. Interestingly, the slope _1/2reoxygenation_ was 3-fold higher in VAS than GAS muscle (i.e., 69 vs. 21%), indicting higher capacities for reperfusion in the knee extensor than ankle extensor muscle.

The observed interaction effect between the aerobic fitness status × ACE-I/D genotype for the slope of deoxygenation and trend for such an effect on SmO_2_ min in the leg muscles during ramp-incremental exercise (Table 2, Figure 3) meets the expectation emanating from the aerobic fitness-associated influence of the ACE-I/D gene polymorphism on mitochondrial volume density (van Ginkel et al., 2015). In this article, we had reported that the volume density of mitochondria in *musculus vastus lateralis* is elevated in aerobically fit non-carriers compared to carriers of the *ACE* D-allele (i.e., endurance athletes) (van Ginkel et al., 2015). As well we had identified that the increase in the volume density of subsarcolemmal mitochondria in *musculus vastus lateralis* with cycling endurance training is amplified in non-carriers compared to carriers of the D-allele (Vaughan et al., 2013). As the content of mitochondria sets the capacity for muscle oxygen consumption, it appears reasonable to expect that the capacity for oxygen consumption, and, conversely, the reduction in muscle oxygen saturation during exercise (Ryan et al., 2014), was largest in the knee extensor muscles of the aerobically fit subjects that did not carry the *ACE* D-allele.

By contrast, we did not find an aerobic fitness-associated interaction effect of the ACE-I/D-genotype on parameters of muscle reoxygenation in *musculus vastus lateralis* during ramp-incremental exercise (Table 2). The ACE-I/D genotype has been found to be associated with altered capillary perfusion of distal limbs (i.e., the fingers) during exhaustive pedaling leg exercise and the capillarization of *Musculus vastus lateralis* (Valdivieso et al., 2017; Fluck et al., 2019) in not-specifically endurance-trained subjects. We observed however a trend (*p* = 0.10) for an interaction between ACE-I/D genotype and fitness state for Δ1/2 reoxygenation, which resolved for VAS in unfit subjects to a trend (*p* = 0.07) for a 10.7% higher Δ_1/2reoxygenation_ in carriers compared to non-carriers of the D-allele (Supplementary Table 3). As well we observed a shallower slope of muscle deoxygenation during ramp-incremental exercise in unfit non-carriers compared to unfit D-allele carriers. This observation may in addition to differences in mitochondria respiration be reflective of the before mentioned better capillary perfusion of D-allele non-carriers during strenuous exercise, overriding of angiotensin 2-mediated vasoconstriction (van Ginkel et al., 2015). Concomitantly, our data relate to findings in ACE-DD genotypes on an altered balance between biochemical reactions that replenish and deplete intermediates of the TCA cycle, which releases energy through the oxidation of acetyl-CoA (Mathes et al., 2015). Nevertheless, an increased respiratory capacity of skeletal muscle is understood to contribute in an over-proportional manner to gains in maximal oxygen uptake with endurance training (Hoppeler et al., 1985) and add together with cardiovascular and pulmonary factors to the systemically measurable maximal oxygen uptake (di Prampero, 2003). Intriguingly, although the slope of muscle deoxygenation during exhaustive ramp exercise (i.e., −0.081 vs. −0.034% SmO_2_ s^−1^) and VO_2_ peak (i.e., 54.4 vs. 40.2 mlO_2_ min^−1^ kg^−1^) differed substantially between the aerobically fit and unfit ACE-II genotypes, the slope of muscle deoxygenation did not differ between the aerobically fit and unfit carriers of the *ACE* D-allele, despite a different VO_2_ peak (i.e., 54.9 vs. 39.8 ml O_2_ min^−1^ kg^−1^). Thus, the fitness × genotype-related effects on muscle deoxygenation were not reflected at the level of statistical significance for VO_2_ peak. ACE-I/D genotypes have been found to demonstrate different hemodynamic responses during maximal exercise (Hellsten and Nyberg, 2015). Collectively our results, therefore, support the view that the association between ACE-I/D × fitness state for oxygen saturation in working skeletal muscle may not always manifest in statistically different maximal oxygen uptake due to a lower effect size at the system level and a possibly different contribution of knee extensor muscle between ACE-I/D genotypes to systemic oxygen uptake (Hoppeler et al., 1985; Jones et al., 2002; Flueck et al., 2010; Valdivieso et al., 2017; Williams et al., 2017).

The identified effects should be viewed in terms of the limitations of this study. First, NIRS-based measures only allow to compute values of oxygen concentration from a rather small volume of tissue which may bear the risk of being confounded by subcutaneous tissue material (Richardson et al., 2001; Grassi and Quaresima, 2016; Jones et al., 2017; Perrey and Ferrari, 2018). The recorded values, however, have been reported to be reliable, especially at the moderate exercise intensities used in this investigation, thus allowing to conduct real-time measurements in a non-invasive manner (Grassi and Quaresima, 2016; Crum et al., 2017; Jones et al., 2017; Perrey and Ferrari, 2018). Furthermore, within our investigation, we also identify considerable reductions for the computed levels of oxygen saturation that are in line with the reported larger degrees for muscle deoxygenation and reoxygenation between the aerobically fit and unfit subjects (Brizendine et al., 2013; Perrey and Ferrari, 2018). As well, for the purpose of data interpretation, it needs to be considered that oxygen saturation reflects the difference between oxygen supply and demand, thus providing only an indirect estimate of the possibly larger changes in the flux of oxygen with exercise (Collins et al., 2011; Rosenberry et al., 2019). This may especially come into account with the onset, and offset, of exercise when metabolic processes are not in a steady-state. Also, we note that in order to avoid other vasodilatation-related influences that may camouflage fitness state and genotype effects, we carried out the ramp-incremental exercise in the mere absence of a warm-up or muscle work to avoid the activation of mitochondrial respiration by unloaded contractions (Nioka et al., 1998; Boone et al., 2012; Perrey and Ferrari, 2018). Finally, we note that our observations are based on a rather heterogeneous population of volunteers, which were not matched for physical fitness between the ACE-I/D genotypes. Both the men and women were recruited to achieve the prospectively determined number of observations to reach the statistical significance of effects, although it had been shown that gender affects muscle deoxygenation during incremental ramp exercise (Murias et al., 2013).

## Conclusion

Our measurements resolve that the ACE-I/D genotype affects aerobic fitness state-related differences in muscle oxygen saturation in recruited skeletal muscle during exhaustive pedaling exercise. Our findings corroborate the view that aerobic metabolism in exercised muscle importantly contributes to the variability of the systemically assessed values for aerobic capacity.

## Data Availability Statement

The datasets presented in this study can be found in online repositories. The names of the repository/repositories and accession number(s) can be found in the article/Supplementary Materials.

## Ethics Statement

The studies involving human participants were reviewed and approved by Ethics Committee of University of Zürich. The patients/participants provided their written informed consent to participate in this study.

## Author Contributions

DN, WF, and MF: study design. MF: funding. MVF, SR, AF, SC, and WF performed experiments. MVF, AF, WP, SC, and MF analyzed experiments. AF, WP, and MF analyzed the data. BG, DN, and MF interpreted the results. BG, MVF, and MF drafted the manuscript. BG, SR, MVF, DN, and MF revised the manuscript. All authors contributed to the article and approved the submitted version.

## Funding

This study was supported by the Swiss Heart Foundation and the RESORTHO Foundation.

## Conflict of Interest

The authors declare that the research was conducted in the absence of any commercial or financial relationships that could be construed as a potential conflict of interest.

## Publisher's Note

All claims expressed in this article are solely those of the authors and do not necessarily represent those of their affiliated organizations, or those of the publisher, the editors and the reviewers. Any product that may be evaluated in this article, or claim that may be made by its manufacturer, is not guaranteed or endorsed by the publisher.

## Supplementary Material

Supplementary Figure 1 Muscle-type dependent differences in reoxygenation after exercise. Box Whisker plots showing the Δ1/2 reoxygenation **(A)**, SmO_2_ max **(B)** and SmO_2_ overshoot **(C)** in VAS and GAS. Lines connect conditions demonstrating significant differences at ^***^*p* < 0.001. ANOVA for the factor “aerobic fitness” with *post-hoc* test of least significant difference.

Supplementary Figure 2 Aerobic fitness × angiotensin-converting enzyme-insertion/deletion (ACE-I/D) genotype associated differences in deoxygenation in leg muscles. Box Whisker plots for the Δ_deoxgenation **(A)** and the slope of deoxygenation **(B)** in VAS and GAS in the function of the aerobic fitness status and the ACE-I/D genotype. Lines connect conditions demonstrating significant differences at ^*^*p* < 0.05 and ^**^*p* < 0.01. ANOVA for the factor “aerobic fitness” with *post-hoc* test of least significant difference.

Supplementary Table 1 Prospective power analysis. Calculation of the prospectively required number of biological replica to reveal statistically significant associations.

Supplementary Table 2 Association of variability is assessed parameters with sex. List of the p-values and effect sizes of the MANOVA for fitness state × genotype × sex. nc, not computable.

Supplementary Table 3 Muscle deoxygenation and re-oxygenation in both studied leg muscles for each genotype and fitness status. List of the average/median (+ SE) values of the MANOVA for fitness state × genotype × muscle type.

Supplementary Table 4 Retrospective power analysis of the observed effects.
