# Supplementary figures and images for: Accelerated Muscle Deoxygenation in Aerobically Fit Subjects During Exhaustive Exercise Is Associated With the ACE Insertion Allele
Source: Front Sports Act Living. 2022 Feb 28;4:814975. doi: 10.3389/fspor.2022.814975 (PMC8918772; doi:10.3389/fspor.2022.814975)

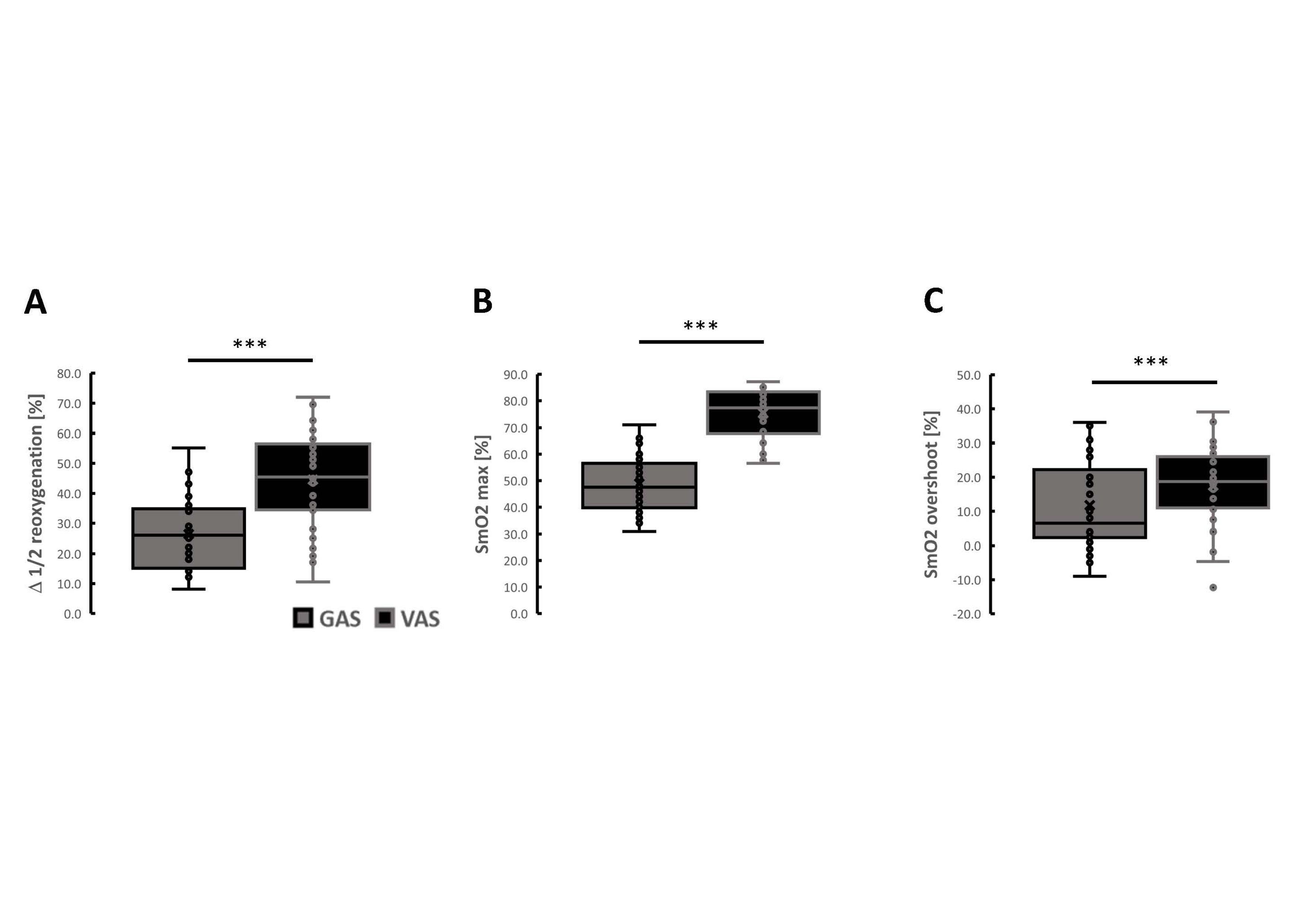

Supplement: Supplementary Figure 1 — Muscle-type dependent differences in reoxygenation after exercise. Box Whisker plots showing the Δ1/2 reoxygenation (A), SmO2 max (B) and SmO2 overshoot (C) in VAS and GAS. Lines connect conditions demonstrating significant differences at ***p < 0.001. ANOVA for the factor “aerobic fitness” with post-hoc test of least significant difference. [file Image_1.JPEG]

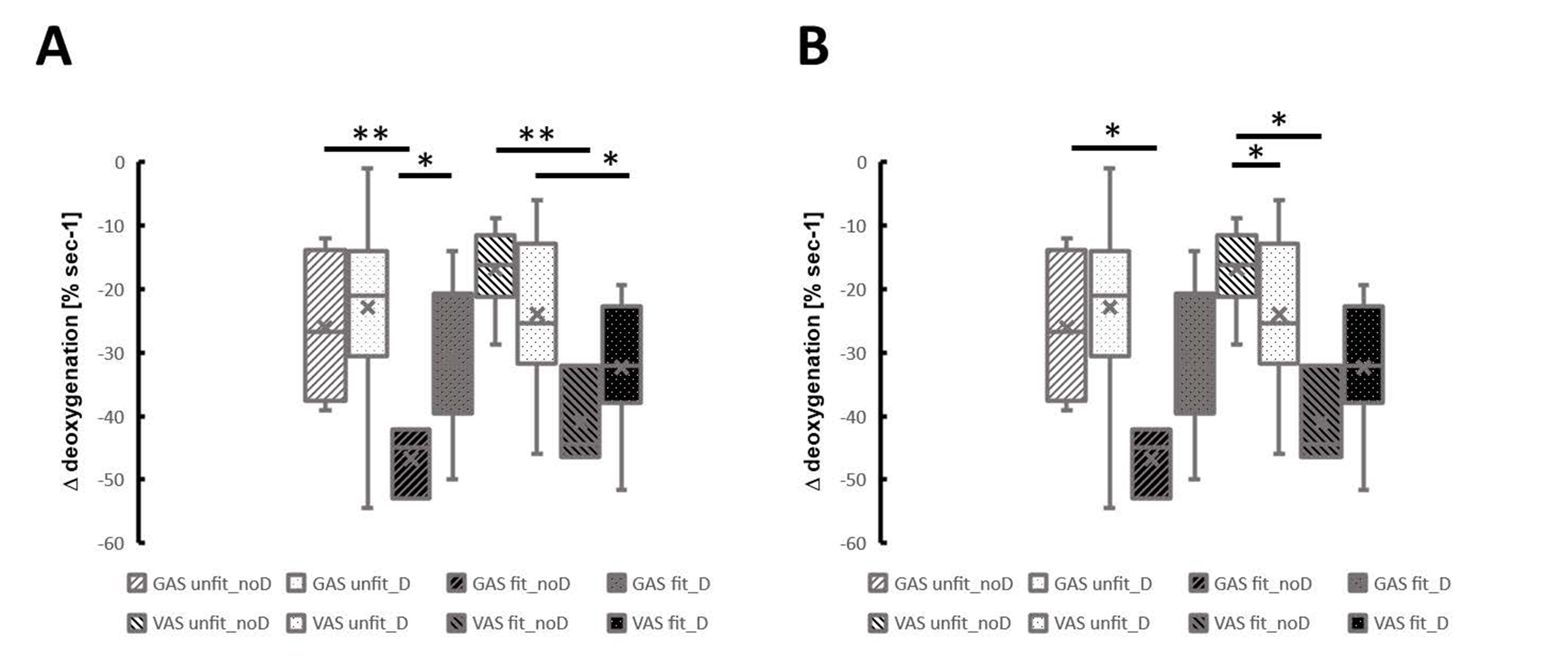

Supplement: Supplementary Figure 2 — Aerobic fitness × angiotensin-converting enzyme-insertion/deletion (ACE-I/D) genotype associated differences in deoxygenation in leg muscles. Box Whisker plots for the Δ_deoxgenation (A) and the slope of deoxygenation (B) in VAS and GAS in the function of the aerobic fitness status and the ACE-I/D genotype. Lines connect conditions demonstrating significant differences at *p < 0.05 and **p < 0.01. ANOVA for the factor “aerobic fitness” with post-hoc test of least significant difference. [file Image_2.JPEG]
