# Supplementary material for: Accelerated Muscle Deoxygenation in Aerobically Fit Subjects During Exhaustive Exercise Is Associated With the ACE Insertion Allele
Source: Front Sports Act Living. 2022 Feb 28;4:814975. doi: 10.3389/fspor.2022.814975 (PMC8918772; doi:10.3389/fspor.2022.814975)
Supplement: Supplementary Table 1 — Prospective power analysis. Calculation of the prospectively required number of biological replica to reveal statistically significant associations. [file Table_1.docx]

**Supplemental table 1: *Prospective power analysis.*** Calculation of the prospectively required number of biological replica to reveal statistically significant associations

***parameter groups effect size (ACE-I/D genotype) prospective n references URL***

mitochondrial volume density unfit vs fit 0.849 15 Flück et al, 2019 https://www.ncbi.nlm.nih.gov/pmc/articles/PMC6518954/

mitochondrial volume density unfit vs fit ACE-II 1.494 6 Flück et al, 2019 https://www.ncbi.nlm.nih.gov/pmc/articles/PMC6518954/

mitochondrial volume density unfit vs fit ACE-ID 1.36 8 Flück et al, 2019 https://www.ncbi.nlm.nih.gov/pmc/articles/PMC6518954/

mitochondrial volume density unfit vs fit ACE-DD 1.00 10 Flück et al, 2019 https://www.ncbi.nlm.nih.gov/pmc/articles/PMC6518954/

total mitochondrial volume density unfit vs fit ACE-I allele 0.073 1240 Vaughan et al. 2013 https://www.ncbi.nlm.nih.gov/pmc/articles/PMC3677975/

subsarcolemmal mitochondrial volume density unfit vs fit ACE-I allele 0.152 292 Vaughan et al. 2013 https://www.ncbi.nlm.nih.gov/pmc/articles/PMC3677975/

subsarcolemmal mitochondrial volume density unfit vs fit ACE-I allele 0.314 70 Vaughan et al. 2013 https://www.ncbi.nlm.nih.gov/pmc/articles/PMC3677975/

VO2max unfit vs fit 0.652 24 Vaughan et al. 2016 https://www.ncbi.nlm.nih.gov/pmc/articles/PMC4794249/

VO2max kg-1 unfit vs fit 1.201 12 Vaughan et al. 2016 https://www.ncbi.nlm.nih.gov/pmc/articles/PMC4794249/
