# Supplementary material for: Accelerated Muscle Deoxygenation in Aerobically Fit Subjects During Exhaustive Exercise Is Associated With the ACE Insertion Allele
Source: Front Sports Act Living. 2022 Feb 28;4:814975. doi: 10.3389/fspor.2022.814975 (PMC8918772; doi:10.3389/fspor.2022.814975)
Supplement: Supplementary Table 2 — Association of variability is assessed parameters with sex. List of the p-values and effect sizes of the MANOVA for fitness state × genotype × sex. nc, not computable. [file Table_2.docx]

**Supplemental table 2:** *Association of variability is assessed parameters with sex.* List of the p-values and effect sizes of the MANOVA for fitness state x genotype x sex. nc, not computable.

***fitness***

***fitness fitness genotype x genotype***

***fitness genotype sex x genotype x sex x sex x sex***

***parameter*** ***p-value h2 p-value h2 p-value h2 p-value h2 p-value h2 p-value h2 p-value h2***

VO2 kg-1 <0.001 0.500 0.682 0.006 0.509 0.016 0.236 0.052 0.650 0.008 0.067 0.119 nc <0.001

VO2 0.002 0.302 0.467 0.020 0.055 0.130 0.249 0.049 0.861 0.001 0.073 0.114 nc <0.001

PPO 0.001 0.365 0.274 0.044 0.003 0.287 0.379 0.029 0.662 0.007 0.208 0.058 nc <0.001

weight 0.114 0.090 0.038 0.150 <0.001 0.376 0.804 0.002 0.660 0.007 0.828 0.002 nc <0.001

height 0.476 0.019 0.911 0.000 0.016 0.195 0.634 0.009 0.834 0.002 0.141 0.078 nc <0.001

BMI 0.007 0.239 0.004 0.265 0.004 0.273 0.393 0.027 0.551 0.013 0.268 0.045 nc <0.001

age 0.743 0.004 0.579 0.012 0.350 0.032 0.985 0.000 0.697 0.006 0.734 0.004 nc <0.001

SmO2_baseline 0.005 0.121 0.099 0.044 0.124 0.038 0.066 0.054 0.116 0.040 0.102 0.043 nc <0.001

SmO2_min 0.005 0.123 0.304 0.017 0.325 0.016 0.310 0.017 0.731 0.002 0.874 <0.001 nc <0.001

Δ_deoxygenation_ <0.001 0.282 0.539 0.006 0.583 0.005 0.017 0.091 0.100 0.044 0.120 0.039 nc <0.001

t_deoxygenation_ <0.001 0.219 0.038 0.069 0.014 0.094 0.950 <0.001 0.379 0.013 0.175 0.030 nc <0.001

slope_deoxygenation_ 0.035 0.071 0.565 0.005 0.607 0.004 0.023 0.082 0.095 0.045 0.305 0.017 nc <0.001

SmO2_max 0.129 0.037 0.799 0.001 1.000 <0.001 0.269 0.020 0.920 <0.001 0.158 0.032 nc <0.001

Δ_reoxygenation_ 0.002 0.144 0.804 0.001 0.481 0.008 0.085 0.048 0.824 0.001 0.094 0.045 nc <0.001

T1/2 _reoxygenation_ 0.046 0.064 0.996 0.000 0.038 0.068 0.183 0.029 0.390 0.012 0.375 0.013 nc <0.001

slope_reoxygenation_ 0.006 0.119 0.797 0.001 0.156 0.033 0.048 0.062 0.016 0.091 0.177 0.030 nc <0.001

SmO2_overshoot 0.927 <0.001 0.439 0.010 0.323 0.016 0.940 <0.001 0.261 0.021 0.569 0.005 nc <0.001
