# Supplementary material for: Accelerated Muscle Deoxygenation in Aerobically Fit Subjects During Exhaustive Exercise Is Associated With the ACE Insertion Allele
Source: Front Sports Act Living. 2022 Feb 28;4:814975. doi: 10.3389/fspor.2022.814975 (PMC8918772; doi:10.3389/fspor.2022.814975)
Supplement: Supplementary Table 3 — Muscle deoxygenation and re-oxygenation in both studied leg muscles for each genotype and fitness status. List of the average/median (+ SE) values of the MANOVA for fitness state × genotype × muscle type. [file Table_3.docx]

**Supplemental table 3:** *Muscle deoxygenation and re-oxygenation in both studied leg muscles for each genotype and fitness status.* List of the average/median (+ SE) values of the MANOVA for fitness state x genotype x muscle type.

**muscle n SmO_2_ baseline SmO_2_ min Δ_reoxygenation_ t_deoxygenation_ slope_deoxygenation_ SmO2_max Δ_reoxygenation_ t1/2 _reoxygenation_ slope_reoxygenation_ SmO_2_ overshoot**

GAS unfit D-allele non-carriers 6 47.8 ± 8.5 27.2 ± 9.0 -20.7 ± 13.5 516.5 ± 139.6 -0.041 ± 0.028 47.8 ± 8.5 24.5 ± 12.5 37.3 ± 22.9 0.483 ± 0.348 -1.5 ± 9.3

GAS unfit D-allele carriers 14 49.0 ± 7.9 29.6 ± 8.3 -19.5 ± 11.5 425.2 ± 102.7 -0.046 ± 0.027 49.0 ± 7.9 22.6 ± 10.7 31.0 ± 29.5 0.664 ± 0.554 -0.8 ± 10.6

GAS aerobically fit D-allele non-carriers 3 63.3 ± 10.7 18.7 ± 3.5 -44.7 ± 7.4 586.7 ± 79.3 -0.078 ± 0.024 63.3 ± 10.7 39.7 ± 13.4 27.2 ± 7.9 0.760 ± 0.249 -7.0 ± 16.5

GAS aerobically fit D-allele carriers 11 51.4 ± 6.6 24.0 ± 9.3 -27.4 ± 13.0 543.8 ± 126.3 -0.050 ± 0.024 51.4 ± 6.6 28.5 ± 10.2 25.1 ± 19.7 0.845 ± 0.689 -2.4 ± 8.4

VAS unfit D-allele non-carriers 6 60.7 ± 9.5 43.9 ± 6.4 -16.8 ± 6.8 497.7 ± 143.8 -0.034 ± 0.012 60.7 ± 9.5 29.2 ± 12.4 40.7 ± 25.3 0.442 ± 0.236 12.4 ± 8.9

VAS unfit D-allele carriers 14 55.2 ± 10.2 31.4 ± 8.6 -23.8 ± 11.1 401.4 ± 104.5 -0.063 ± 0.033 55.2 ± 10.2 39.9 ± 16.0 37.4 ± 16.0 0.628 ± 0.352 16.1 ± 12.5

VAS aerobically fit D-allele non-carriers 3 65.8 ± 16.6 24.8 ± 9.2 -41.0 ± 7.8 506.0 ± 77.5 -0.081 ± 0.014 65.8 ± 16.6 57.7 ± 5.7 30.7 ± 4.2 0.960 ± 0.211 16.7 ± 12.2

VAS aerobically fit D-allele carriers 11 58.0 ± 9.3 24.7 ± 5.4 -33.3 ± 10.6 554.0 ± 114.3 -0.060 ± 0.017 58.0 ± 9.3 54.9 ± 8.1 36.7 ± 8.8 0.777 ± 0.154 21.6 ± 11.2
